# Differential In Vitro Growth and Cell Killing of Cancer versus Benign Prostate Cells by Oncolytic Parainfluenza Virus

**DOI:** 10.3390/pathogens11050493

**Published:** 2022-04-21

**Authors:** Kritika Kedarinath, Griffith D. Parks

**Affiliations:** Burnett School of Biomedical Sciences, College of Medicine, University of Central Florida, Orlando, FL 32827, USA; kritika.kedarinath@knights.ucf.edu

**Keywords:** oncolytic virus, parainfluenza virus, cell killing

## Abstract

The development of effective oncolytic viruses will require understanding the differences in virus replication and killing between normal and cancer cells. Here, we have evaluated infections of metastatic cancer (22Rv1) and benign non-tumorigenic (BPH-1) prostate cell lines with a mutant parainfluenza virus 5 (P/V/F) encoding a defective V protein and a hyperfusogenic F protein. Under low multiplicity of infection (MOI), the P/V/F mutant efficiently spread in 22Rv1 cells but was restricted in BPH-1 cells due to type-I interferon (IFN-I) responses. In mixed co-cultures, the P/V/F mutant showed specificity towards and spread within the 22Rv1 cells versus BPH-1 cells. Under high MOI conditions, both BPH-1 and 22Rv1 cells showed efficient infection by the P/V/F mutant. However, compared to BPH-1 cells, the 22Rv1 cancer cells showed increased cytopathic effect, higher induction of caspase-8 and -9, and extensive syncytia formation. In 22Rv1 spheroid cultures, P/V/F infection was less efficient compared to monolayers, but the virus was able to spread through spheroids and induce death. These data indicate that IFN-I sensitivity is a major determinant of specificity of P/V/F spread through populations of cancer versus benign cells, and additionally, differences in activation of apoptotic pathways and syncytia formation can contribute to differential outcomes in cancer versus benign cells.

## 1. Introduction

Prostate cancer is the second most prevalent cancer and the second leading cause of cancer-related deaths in American men. According to the American Cancer Society, 1 in 8 men will be diagnosed with prostate cancer during their lifetime [1]. Typically, treatments for benign stage prostate cancer are successful. However, aggressive tumors are formed when cancer progresses into its advanced stages, which are resistant by nature to a number of treatments. There is a growing interest in the use of oncolytic viruses as therapeutic agents for prostate cancer. Whether they are engineered or have inherent properties, these infectious organisms are intended to overcome one of the main hurdles of cancer therapy: targeting tumor cells while leaving the healthy/normal cells unaffected. The advent of oncolytic viruses has been fueled by the recent approval of IMLYGIC™ (talimogene laherparepvec) by the Food and Drug Administration, which is a genetically modified herpes simplex virus type 1 that can be used to treat melanoma tumors [2]. The advantages of oncolytic viral therapy include: that viruses can be modified to replicate to high titers in cells that have an abnormal cellular environment or disrupted signaling pathways [3]; they target the entire cell, as opposed to some specific drugs which target isolated cellular pathways within the cell [4]; they are capable of generating immune responses that can aid in killing or clearing the tumor from the host. These immune responses can also contribute to the specificity of the vectors towards tumor cells instead of normal cells [5,6,7,8]. 

One such virus-induced immune response is the type-I interferon (IFN-I) pathway. This potent system consists of a production phase and a signaling phase. IFN-I is produced from cells when viral signals such as double-stranded RNA are produced, which are then sensed by viral sensors like Retinoic acid-inducible gene-I (RIG-I) and melanoma differentiation-associated protein-5 (MDA-5), leading to transcription of IFN-I genes, followed by synthesis and subsequent secretion from the cell [9]. Once secreted, IFN-I can bind through autocrine or paracrine means to their receptors at the cell surface, which stimulates signal transducer and activator of transcription (STAT) dependent signaling pathways that ultimately result in the transcriptional induction of a wide range of different interferon-stimulated genes (ISGs). Many of these ISGs can act at various stages of the viral infection cycle to inhibit viral infection and/or replication in a host cell [9]. Many cancer cells develop defects in IFN-I production or resistance to IFN-I signaling, and it is thought this reflects the need to bypass the anti-proliferative effects of IFN-I [10,11]. It has been proposed that these defects in IFN-I sensitivity can be exploited with viral vectors that can replicate in IFN-I defective tumor cells, but not in IFN-I competent normal cells [11]. This raises the importance of understanding how normal and cancer cells respond to different oncolytic viruses and the role that IFN-I responses play in this specificity. 

Some viruses from the *Paramyxoviridae* family show great potential as oncolytic viruses, including mumps virus, Newcastle disease virus, and measles virus, which has been developed for various cancer therapies [12,13,14,15,16,17]. Wild type (WT) parainfluenza virus 5 (PIV5) has been shown to be largely non-cytopathic in nature in most human cell types in vitro. WT PIV5 encodes a V protein in the P/V gene which is capable of blocking IFN-I production through inhibition of MDA5 activation [9]. V protein is also able to block IFN-I signaling by targeting STAT1, ultimately leading to its degradation [18,19,20,21]. Thus, WT PIV5 appears to be inappropriate for development as an oncolytic virus. However, we have shown that introducing six amino acid (AA) substitutions in the P/V gene converts WT PIV5 into a mutant (P/V mutant) which kills tumor cells, induces IFN-I, and is unable to block IFN-I signaling [22,23,24,25,26,27]. 

**Figure 1:**
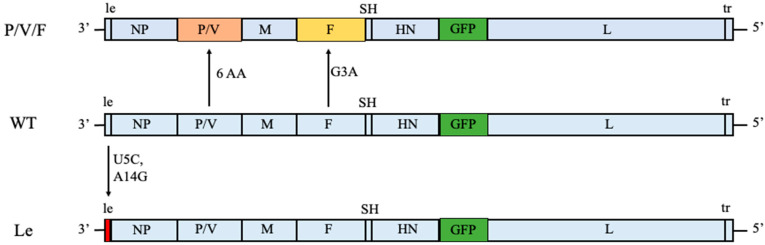
Genome structures of PIV5 WT and mutant viruses. Schematic representation of PIV5 WT, P/V/F and Le mutant genomes, with the addition of GFP gene between HN and L, as previously described [29]. Six amino acid (AA) substitutions in the P/V gene and a single point G3A mutation in F gene of WT resulted in the generation of the P/V/F mutant. Nucleotide substitutions in the 5th and 14th positions of the leader promoter (U5C and A14G mutations) resulted in the Le mutant. Note that the Le mutant has an intact P/V gene.

In this study, we have expanded our analysis of PIV5 as an oncolytic virus to include two other PIV5 mutant viruses. As shown in Figure 1, we previously generated the P/V/F mutant which contains the same alterations to the P/V gene as described above, but has an additional mutation in the viral fusion protein F. Here, a single Gly-to-Ala substitution in the fusion peptide of the F protein results in a mutant virus with elevated hyperfusogenic properties [28]. This hyperfusogenic P/V/F mutant is capable of inducing cytopathic effects in tumor cells by syncytia formation and is also able to reduce prostate tumor burden in vivo [28]. However, the behavior of this PIV5 mutant in normal or benign cells has not been examined. As a second alternative, our prior work showed that two nucleotide changes in a highly conserved 3′ genomic element (U5C and A14G) of the leader (Le) promoter in the WT genome resulted in a mutant (Le mutant) which overexpressed viral proteins and mRNAs and was also capable of inducing killing of lung tumor cells [27] (Figure 1). Both the P/V/F and Le mutants showed elevated induction of IFN-I. 

Here, we have analyzed the properties of the P/V/F and Le mutant PIV5 viruses as potential oncolytic viruses, defining their growth and cytopathic properties in cancer versus benign prostate cells in vitro. This includes the role of IFN-I in selective spread through cell populations, as well as non-IFN-I factors in benign prostate and cancer cell lines that can contribute to the killing of cancer cells.

## 2. Results

### 2.1. Differential Spread and Growth Properties of PIV5 WT and Mutant Viruses in Prostate Cancer and Benign Cell Lines

**Figure 2:**
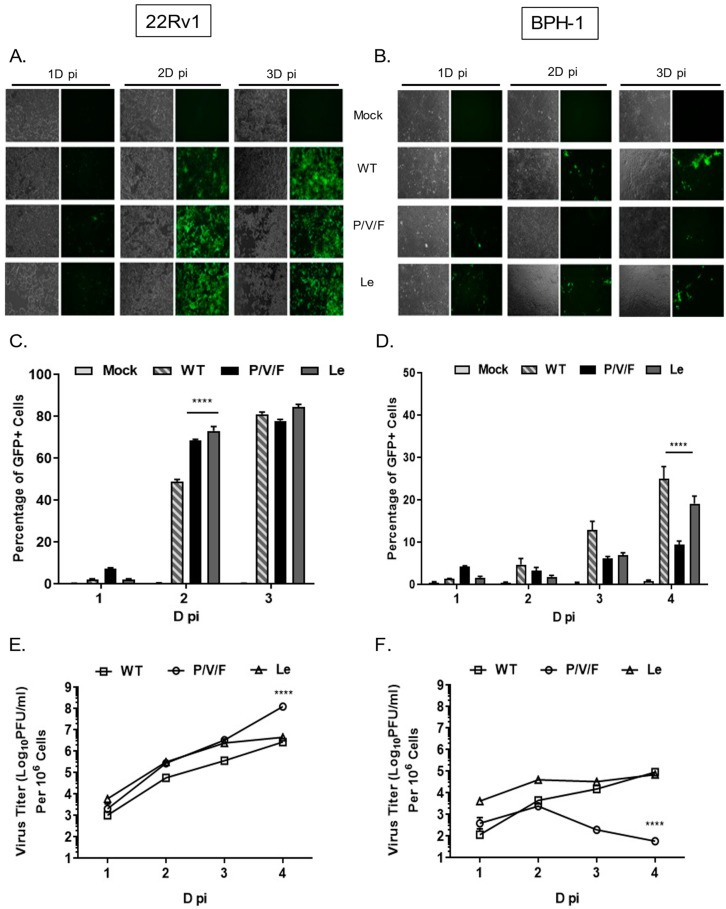
Spread and growth of PIV5 WT and mutant viruses in prostate cancer and benign cells. 22Rv1 (**A**,**C**,**E**) and BPH-1 cells (**B**,**D**,**F**) were either mock-infected or infected with control WT virus or the indicated mutant PIV5 viruses at a MOI of 0.05. At the indicated time points, bright field (BF) and fluorescence (FL) microscopy images were taken (**A**,**B**) and GFP+ cells were quantified using flow cytometry (**C**,**D**). To determine multi-step growth properties, 22Rv1 (**E**) and BPH-1 (**F**) cells were infected with WT virus at a MOI of 0.01 or indicated mutants at a MOI of 0.05. Media from infected cells were harvested at the indicated time points and titers of infectious virus units were determined by plaque assays (**E**,**F**). Viral titers were normalized to 10^6^ cells and expressed as PFU/mL on the logarithmic scale. Values are the mean of three samples, with error bars representing standard deviation., **** indicates *p*-value < 0.0001.

22Rv1 is a human prostate epithelial carcinoma cell line, which is derived from a xenograft mouse model after castration-induced regression and relapse. BPH-1 is an immortalized benign prostate hyperplasia cell line established from primary prostate tissue and is non-tumorigenic in nature. WT and mutant PIV5 viruses have been engineered to encode GFP as an additional gene between the viral HN and L genes [29]. To determine the ability of PIV5 WT and mutant viruses to spread through a population of benign prostate cells (BPH-1) and prostate cancer cells (22Rv1), monolayers of cells were mock-infected or infected at a low multiplicity of infection (MOI) of 0.05 with PIV5 WT, P/V/F mutant or Le mutant. Bright field (BF) and fluorescence images (FL) were taken 1, 2, and 3 days (D) post-infection (pi), and virus-infected cells were quantified by flow cytometry. As shown in Figure 2A, WT and mutant viruses showed a time-dependent spread through most of the 22Rv1 cell population by 3D pi. Quantification by flow cytometry showed that ~80% of infected 22Rv1 cells were GFP+ by 3D pi for all three viruses (Figure 2C). By contrast, infected BPH-1 cells showed only low levels of GFP+ cells over time, with more WT and Le virus-infected cells positive at 3D pi compared to that of a P/V/F mutant (Figure 2B). Flow cytometry quantification showed that ~20% of the BPH-1 cells were GFP+ when infected with either WT or Le mutant virus, compared to only ~10% of the cells infected with the P/V/F mutant by 4D pi (Figure 2D). 

Multi-step growth properties of WT and mutant viruses in 22Rv1 and BPH-1 cells were determined by infecting with control WT virus at a MOI of 0.01, while P/V/F and Le mutants infections were carried out at a MOI of 0.05. Media from infected cells were harvested at indicated time points shown in Figure 2E,F, and virus titers were determined by plaque assay. In 22Rv1 cancer cells, growth of the P/V/F and Le mutant viruses was to slightly higher titers than PIV5 WT, with the P/V/F mutant growing to ~2 logs higher in titer than the other viruses by 4D pi. By contrast, in BPH-1 benign cells, growth of the P/V/F mutant peaked at a titer slightly higher than 10^3^ PFU/mL by 2D pi but then was reduced to the limit of detection at later time points (Figure 2F). PIV5 WT and Le mutant grew to similar titers, which were ~10^3^ fold higher than the P/V/F mutant. All viral titers from BPH-1 cells across the time course were lower compared to corresponding titers observed in the 22Rv1 cells (Figure 2E). Taken together, these data indicate that the WT and mutant viruses are capable of comparable growth in and spread through a population of 22Rv1 cells, but the P/V/F mutant is restricted in growth in BPH-1 cells relative to WT or Le mutant viruses. 

The below data indicate that WT and Le mutant viruses are able to grow and spread through a population of benign prostate cells, indicating their limitations as potential candidate oncolytic viruses with specificity for tumor versus normal cells. The higher degree of relative restriction in BPH-1 benign cells, seen with the P/V/F mutant, supports this virus as a promising oncolytic virus to study further. In the subsequent studies described below, we have focused on characterizing the restriction and oncolytic potential of the P/V/F mutant in BPH-1 and 22Rv1 cell lines.

### 2.2. IFN-I Production, and Not Inherent Replication Defects, Restrict P/V/F Infection in Benign Prostate Cells

**Figure 3:**
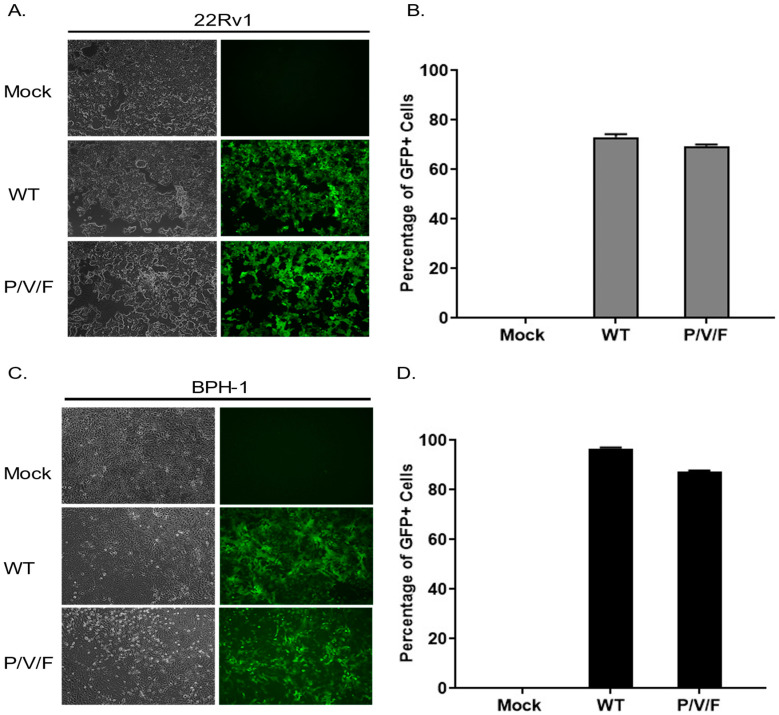
Restricted spread of the P/V/F mutant virus in benign prostate cancer cells is not due to the inherent inability to infect cells. 22Rv1 (**A**,**B**) and BPH-1 cells (**C**,**D**) were either mock-infected or infected with WT or P/V/F mutant at a MOI of 10. At 1D pi, BF and FL microscopy images were taken (**A**,**C**) and GFP+ cells were quantified using flow cytometry (**B**,**D**). Values are the mean of three samples, with error bars representing standard deviation.

We hypothesized that the restriction of low multiplicity P/V/F mutant growth in the BPH-1 cells was due to either an inherent restriction in the capacity of these cells to support viral replication or in the triggering of anti-viral responses which could prevent viral spread through the population of cells. To test the former hypothesis, 22Rv1 and BPH-1 cells were mock-infected or infected at a high MOI of 10 with WT (as a control) or P/V/F mutant, and BF and FL microscopy images were taken at 1D pi. As shown in Figure 3, both the WT and P/V/F mutant were able to infect most of the cell population of both 22Rv1 (Figure 3A) as well as BPH-1 (Figure 3C) cells. Quantification of GFP+ cells using flow cytometry showed that ~60–70% of the 22Rv1 cells (Figure 3B) and ~80–90% of BPH-1 cells (Figure 3D) were infected by either the WT or P/V/F mutant. These data indicate that there is no inherent restriction in the capacity of the BPH-1 cells to support P/V/F infection. 

**Figure 4:**
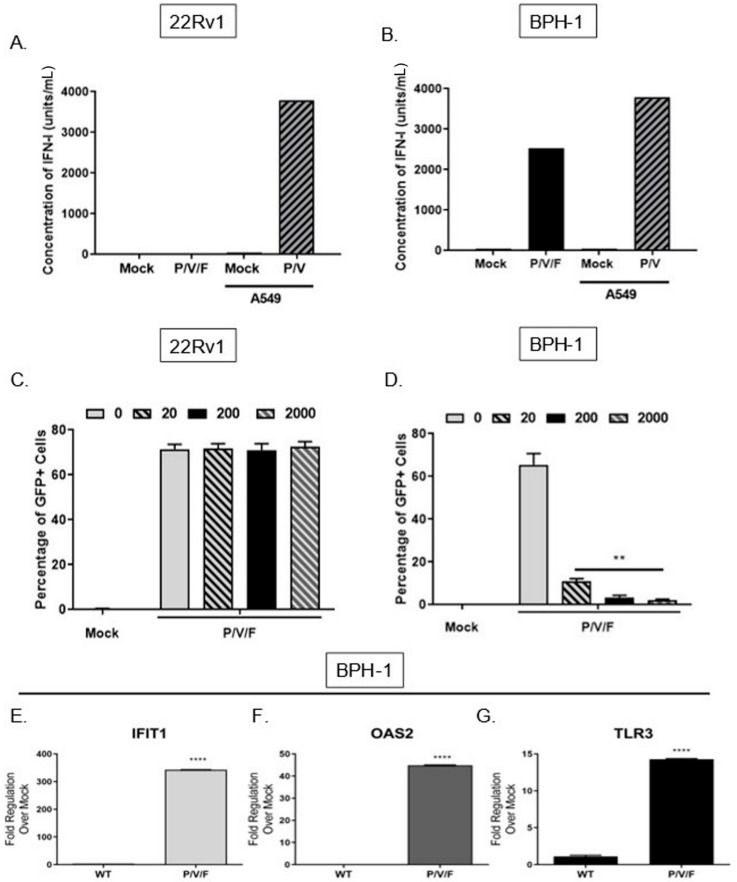
Differential IFN-I production and signaling in prostate cancer and benign cells upon P/V/F mutant infection. 22Rv1 (**A**) and BPH-1 cells (**B**) were either mock-infected or infected at a MOI of 10 with P/V/F mutant. Levels of IFN-I were quantified using a biological detection assay, as described in Materials and Methods. Mock and P/V mutant-infected A549 cells were used as negative and positive controls, respectively. Data is a representation of two separate experiments. 22Rv1 (**C**) and BPH-1 (**D**) cells were pretreated with indicated concentrations of universal IFN-I (units/mL) overnight, followed by infection at MOI of 10 with the P/V/F mutant for 24 h. GFP+ cells were then quantified using flow cytometry. (**E**–**G**) BPH-1 cells were either mock-infected or infected overnight with P/V/F mutant at a MOI of 10 and total cellular RNA was isolated and used to determine relative expression levels of IFIT1 (**E**), OAS2 (**F**) and TLR3 (**G**) via quantitative RT-PCR. Data is a representation of two separate experiments. Values are the mean of three samples, with error bars representing standard deviation. ** and **** indicating *p*-values < 0.002 and <0.0001, respectively.

We tested the alternative hypothesis that restricted low MOI growth of the P/V/F mutant in BPH-1 cells was due to activation of an anti-viral response, particularly IFN-I. To quantify the production of IFN-I in response to P/V/F mutant infection, 22Rv1 and BPH-1 cells were mock-infected or infected at a MOI of 10 with P/V/F mutant. As a control, IFN-I production was assayed from A549 lung carcinoma cells that were mock-infected (negative control) or infected with the P/V mutant at a MOI of 10 (positive control) for 24 h [23]. A high MOI infection was performed to synchronize the infection within the population and to ensure reliable quantification of detectable IFN-I. Extracellular media were then collected, and levels of IFN-I were determined using a biological assay on indicator HEK-Blue™ IFN-α/β cells, as described in Materials and Methods. As shown in Figure 4A, there were no detectable levels of IFN-I produced by mock or P/V/F-infected 22Rv1 cells. By contrast, infection of BPH-1 cells with the P/V/F mutant resulted in ~2500 units/mL of IFN-I (Figure 4B). 

To determine the capacity for differential signaling in response to IFN-I, 22Rv1 and BPH-1, cells were treated for ~14 h with increasing concentrations (units/mL) of universal IFN-I, and then mock-infected or infected with the P/V/F mutant at a MOI of 10 for 24 h. The percentage of GFP+ cells was determined by flow cytometry. As shown in Figure 4C, the percentage of infected 22Rv1 cells remains relatively similar following treatment with increasing concentrations of IFN-I treatment, suggesting that these cells are unresponsive to high concentrations of IFN-I. By contrast, BPH-1 cells showed a dose-dependent decrease in P/V/F mutant infection (GFP+) with increasing concentrations of IFN-I treatment (Figure 4D). 

To confirm that functional IFN-I signaling was occurring during P/V/F mutant infection of BPH-1 cells, expression levels of three key ISGs (IFIT1, OAS2 and TLR3) were quantified. BPH-1 cells were either mock-infected or infected overnight (~14 h) with the WT (as a control) or P/V/F mutant at a MOI of 10, and quantitative RT-PCR was performed. As shown in Figure 4E, the IFIT1 gene was upregulated by ~350 fold upon P/V/F mutant infection, with a much lower induction by the WT infection. As shown in Figure 4F,G, P/V/F mutant infection upregulates expression levels of OAS2 by ~40 fold and TLR3 expression by ~15 fold, respectively, with only modest gene expression changes seen with the WT infection. 

Taken together, these data indicate that the 22Rv1 cells are unable to produce detectable levels of IFN-I after P/V/F infection or create an anti-viral response when exposed to titrated concentrations of IFN-I. In contrast, the BPH-1 cells are strong inducers of IFN-I at a high multiplicity of infection and respond to IFN-I after P/V/F mutant infection, with induction of three key ISGs (OAS2, IFIT1 and TLR3), indicating the establishment of an anti-viral state.

### 2.3. IFN-β Is a Key Contributing Factor to Restriction of P/V/F Mutant Spread through a Population of Benign Prostate Cells

**Figure 5:**
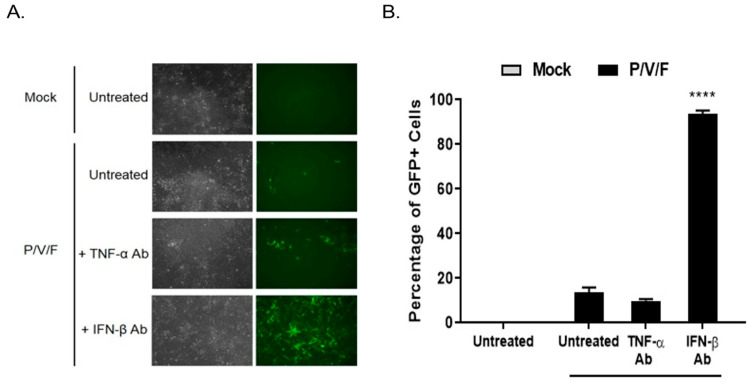
IFN-β is a major determinant of restricted P/V/F growth in benign prostate cells. BPH-1 cells were mock-infected or infected with P/V/F mutant at a low MOI of 0.05. Media were either left untreated or supplemented with antibodies against TNF-α (TNF-α Ab) or IFN-β (IFN-β Ab). At 3D pi, BF and FL images were taken (**A**) and GFP+ cells were quantified using flow cytometry (**B**). Values are the mean of three samples, with error bars representing standard deviation. **** indicating *p*-value < 0.0001.

To determine if IFN-I functionally contributed to the low MOI restriction of P/V/F mutant infection in BPH-1 cells, cells were either mock-infected or infected with the P/V/F mutant at a low MOI of 0.05. Media were then supplemented with or without antibody (Ab) against TNF-α or IFN-β for 3D, and flow cytometry was performed to determine the percentage of GFP+ cells. A low MOI infection was performed to control the infection of P/V/F mutant and to effectively neutralize the secreted levels of IFN-β. As shown in Figure 5A, BF and FL microscopy images showed no visible change in the number of GFP+ cells in the cell population with TNF-α Ab treatment compared to untreated control samples. However, IFN-β Ab treatment resulted in an increase in the number of GFP+ cells. Quantification of infected cells using flow cytometry (Figure 5B) showed that ~90% of the BPH-1 cells appear to be infected (GFP+) after treatment with IFN-β Ab. These data indicate that IFN-β is a major contributing factor to the restriction of the spread of the P/V/F mutant in BPH-1 cells.

### 2.4. Spread of the P/V/F Mutant in Co-Cultures of Prostate Cancer and Benign Cells

**Figure 6:**
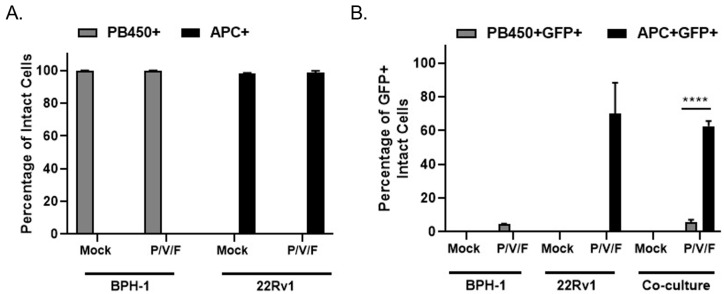
P/V/F mutant spread is limited to prostate cancer cells in mixed cultures of cancer and benign prostate cells. BPH-1 and 22Rv1 cells were pre-stained with CellTrace™ Violet and CellTrace™ Far Red, respectively, and then either seeded alone or together in a co-culture. Stained cells were mock-infected or infected with the P/V/F mutant at a MOI of 0.01. At 3D pi, stained BPH-1 (PB450+) and 22Rv1 (APC+) cells were quantified using flow cytometry in their respective populations (**A**). GFP+ virus-infected and stained BPH-1 (PB450+GFP+) and 22Rv1 (APC+GFP+) cells were also similarly quantified in their respective populations (**B**). Values are the mean of three samples, with error bars representing standard deviation. **** indicating *p*-values < 0.002 and <0.0001, respectively.

A co-culture experiment was carried out to test the hypothesis that P/V/F mutant infection would selectively target 22Rv1 cells even in the presence of benign BPH-1 cells, mimicking an infection at the interface between solid tumor and healthy tissue in vitro. BPH-1 cells were stained with CellTrace™ Violet and 22Rv1 cells were stained with CellTrace™ Far Red (as described in Materials and Methods). To imitate the interface of a tumor and surrounding benign cells, CellTrace™ Violet stained BPH-1 (20,000) and CellTrace™ Far Red stained 22Rv1 (40,000) cells were seeded either alone (as controls) or together in a co-culture. Stained cells were then either mock-infected or infected with the P/V/F mutant at a MOI of 0.01 and flow cytometry was performed in 3D pi, to determine the percentage of stained and infected (GFP+) cells. CellTrace™ Violet stained BPH-1 cells were detected in the PB450 channel (PB450+ cells) while the CellTrace™ Far Red stained 22Rv1 cells were detected in the APC channel (APC+ cells). Figure 6A shows the efficiency of staining mock and P/V/F infected BPH-1 (PB450+) and 22Rv1 (APC+) cells. The data indicate that the mock-infected BPH-1 and 22Rv1 cells had 100% staining when seeded. It was also observed that the percentage of stained BPH-1 (PB450+) and 22Rv1 (APC+) cells do not change the staining efficiency of the respective dyes, after the P/V/F mutant infection. 

To determine if P/V/F infection was restricted to 22Rv1 cells, the percentage of infected GFP+ cells was determined within the individual PB450+ and APC+ stained populations. Flow cytometry showed (Figure 6B) the percentage of stained and infected BPH-1 cells (PB450+GFP+) and 22Rv1 cells (APC+GFP+) that were either seeded alone or together in the co-culture. In the BPH-1 cells seeded alone, less than 10% of the BPH-1 cells were infected by the P/V/F mutant and in the 22Rv1 cells seeded alone, ~70% of the population was infected. These values did not change substantially in the co-culture, since ~10% of the BPH-1 population and ~60% of the 22Rv1 population were GFP+ after infection with the P/V/F mutant. These data support the hypothesis that P/V/F infection is largely restricted to 22Rv1 cells in co-culture. 

Taken together, these data indicate that the P/V/F mutant is restricted to spread in BPH-1 but not 22Rv1 cells, even in the context of a mixed population of cancer and benign cell types and the overpopulation of cancer cells. 

### 2.5. Relative Potential for PIV5 WT and P/V/F Virus Infections to Cause Cell Killing and Cytopathic Effects in Prostate Cancer and Benign Cells

**Figure 7:**
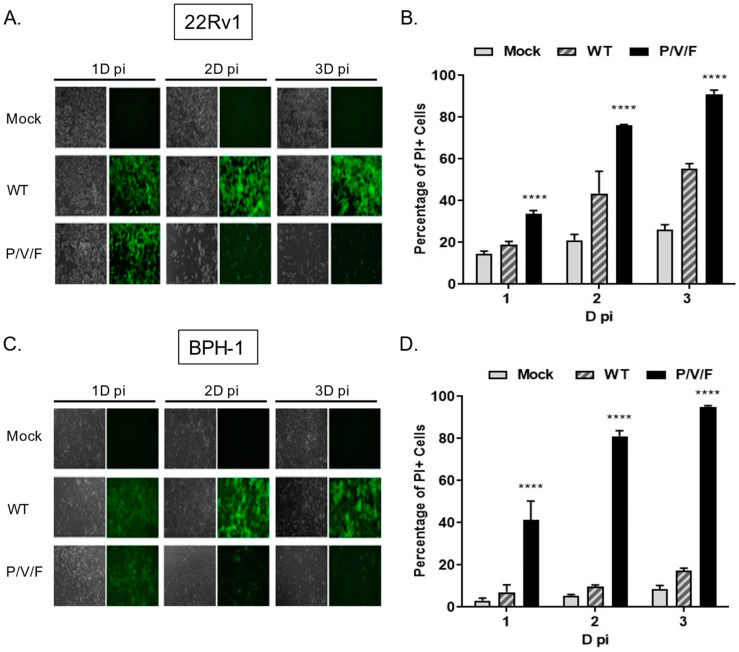
Killing of prostate cancer and benign cells following PIV5 WT and P/V/F mutant infection. 22Rv1 (**A**,**B**) and BPH-1 cells (**C**,**D**) were mock-infected or infected with WT or P/V/F mutant at a MOI of 10. At the indicated time points, BF and FL microscopy images were taken (**A**,**C**) and the percentage of PI+ cells in the population was quantified by flow cytometry (**B**,**D**). Values are the mean of three samples, with error bars representing standard deviation. **** indicating *p*-value < 0.0001.

Given the capacity for the P/V/F mutant to infect and replicate in benign BPH-1 cells, we tested the hypothesis that the P/V/F mutant differed in the ability to kill prostate cancer and benign cells. To test this, 22Rv1 and BPH-1 cells were mock-infected or infected at a MOI of 10 with WT (as a control) or P/V/F mutant viruses. At the times indicated in Figure 7, BF and FL images were captured and cell death was quantified by propidium iodide (PI) stain and flow cytometry. As shown in Figure 7A,C, both 22Rv1 and BPH-1 cells showed bright GFP fluorescence with a minimal loss of cells when infected with the PIV5 WT virus, even at late times post-infection, such as 3D. By contrast, P/V/F infection showed a time-dependent loss of cells in the case of both cell lines, by 2D pi, respectively. To quantify this CPE observed, propidium iodide (PI) stain was utilized to determine the percentage of non-viable cells via flow cytometry (Figure 7B,D). Consistent with this, P/V/F mutant infection of both 22Rv1 and BPH-1 cells showed time-dependent increases in PI staining, from ~35–50% PI+ to >90% PI+ cell populations (black bars, Figure 7B,D). The largest difference in cell killing between 22Rv1 and BPH-1 cells was seen with PIV5 WT infection, which was between ~20% (1D) to 50% (3D) for 22Rv1 cells compared to a very low 5–10% for BPH-1 cells. These data indicate that while restricted at low MOI, the P/V/F mutant exhibits significant cell killing in both 22Rv1 as well as BPH-1 cells at high MOI, whereas the WT virus only induces comparable levels of killing in 22Rv1 cells. To address the cytopathic effect exhibited by infection with the WT and P/V/F mutant at a low MOI, 22Rv1cells were infected at a MOI of 0.05, and flow cytometry was performed to quantify the percentage of PI+ cells at 1, 2 and 3D pi (Supplementary Figure S1). The P/V/F mutant showed a time-dependent increase in cell death, while the WT infection did not exhibit the same trend. This was also seen during infections of primary human keratinocytes cells (Supplementary Figure S2). 

**Figure 8:**
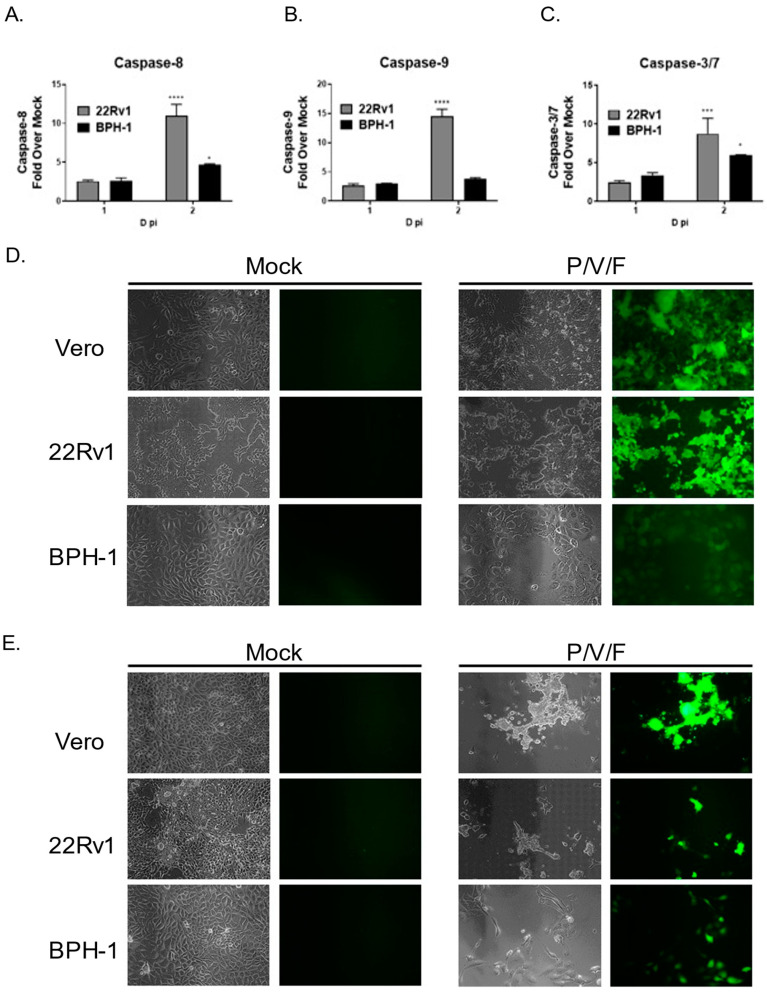
Differential apoptosis and cell-cell fusion in P/V/F mutant-infected prostate cancer and benign cells. Caspase activation, (**A**–**C**). 22Rv1 and BPH-1 cells were either mock-infected or infected with P/V/F mutant at a MOI of 10. At the indicated D pi, Caspase-Glo^®^ Assays specific for caspase-8 (**A**), -9 (**B**), and -3/7 (**C**) were carried out. Values are the mean of three samples, with error bars representing standard deviation. *, ***, and **** indicating *p*-values < 0.033, <0.0002, and <0.0001, respectively. Syncytia formation, (**D**,**E**) 22Rv1 and BPH-1 cells were mock-infected or infected with P/V/F mutant or WT at a MOI of 10. As a control, Vero cells were infected at a MOI of 0.05 with the P/V/F mutant. At 1D pi (**D**) and 2D pi (**E**), BF and FL images (under 20× magnification) were taken to observe syncytia formation.

We tested the hypothesis that cell killing correlated with differences in induction of apoptotic pathways in infected 22Rv1 and BPH-1 cells. Cells were either mock-infected or infected with the P/V/F mutant at a high MOI, and functional levels of caspase-8, -9, and -3/7 were determined at 1D and 2D pi using Caspase-Glo^®^ Assays (as described in the Materials and Methods). Caspase levels were expressed as a fold increase of virus-infected cells over mock-infected cells. As shown in Figure 8A–C, P/V/F-infected 22Rv1 cells show higher induction of caspase-8, -9 and -3/7 by 2D pi, compared to corresponding infected BPH-1 cells. 

The P/V/F mutant contains a single point mutation in the F gene, which results in hyperfusogenic properties, resulting in a cytopathic effect via syncytia formation [28]. We hypothesize that this form of death could be playing a role in the differential cytopathic effects observed. To determine if P/V/F-infected 22Rv1 and BPH-1 cells showed differential syncytia formation, cells were either mock-infected or infected with the P/V/F mutant at a MOI of 10. As a positive control, Vero cells were infected with the P/V/F mutant at a MOI of 0.05, as this has been previously shown to cause syncytia formation [28]. BF and FL images (20×) were taken on 1D (Figure 8D) and 2D (Figure 8E) pi. As shown in Figure 8D,E, P/V/F mutant-infected 22Rv1 cells showed large amounts of syncytia formation, comparable to infected Vero cells. By 2D pi, most of the cell monolayer was no longer intact. In contrast, infected BPH-1 cells do not exhibit overt signs of cell fusion or syncytia formation. These data indicate that the P/V/F mutant induces much higher upregulation of caspases-8, and -9 while a more modest upregulation was observed in caspase-3/7 in infected 22Rv1 cells compared with BPH-1 cells. Additionally, differential syncytia formation was observed in 22Rv1 vs. BPH-1 cells. It appears that the different apoptotic signals (caspase activation and syncytia formation) in these two cell types contribute to cell killing, observed in these two cell lines upon P/V/F mutant infection. 

### 2.6. Cell Killing Properties of the P/V/F Mutant in a Three-Dimensional Prostate Cancer Spheroid Model

**Figure 9:**
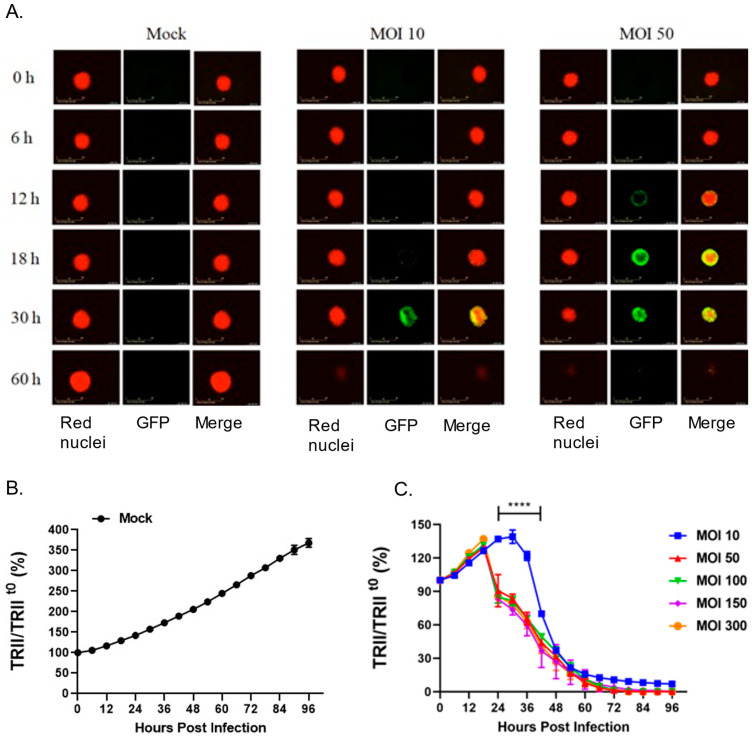
P/V/F mutant killing of infected prostate cancer cells in a three-dimensional spheroid tumor model. Spheroid cultures of 22Rv1-NLR cells were either mock-infected or infected with P/V/F mutant at increasing MOIs of 10, 50, 100, 150, and 300. (**A**) Images (10× magnification) were captured at the indicated time points depicting red (mock) or green (infected) FL in three-dimensional spheroid cultures at MOI of 10 or 50. (**B**,**C**). Red fluorescence (TRII) in cultures of mock (**B**) and infected (**C**) spheroids was recorded and quantified every 6 h and are expressed as a percentage of the initial value at time 0. Values are the mean of three samples, with error bars representing standard deviation. **** indicating *p*-value < 0.0001.

Given that the P/V/F virus is hyperfusogenic [28] and that 22Rv1 cells fuse after P/V/F infection, we hypothesized that three-dimensional spheroid cultures of 22Rv1 cells would be highly sensitive to P/V/F virus due to cell–cell spread and syncytia formation. To test this, 22Rv1 cells were modified to express a nuclear red fluorescent protein (22Rv1-NLR) which can be detected in real-time assays for cell viability. These 22Rv1-NLR cells were then cultured to form spheroids (as described in Materials and Methods), and either mock-infected or infected with P/V/F mutant at increasing MOIs. Real-time images of spheroid cells (red) and virus-infected cells (green) were recorded on an Incucyte instrument every 6 h (Figure 9A) and quantitative analysis of levels of total red integrated intensity (TRII) were measured across the indicated time course (Figure 9B,C). Values of TRII were expressed as a ratio compared to time 0 (TRII/TRII^t0^). 

Figure 9A shows representative snapshots of spheroids (red) and infected cells (green) observed at time points after infection at two different MOIs. Mock-infected spheroids continued to grow in culture and showed an increase in the size of the spheroid and the red fluorescence intensity over time (Figure 9A). The efficiency of P/V/F infection was dependent on MOI. This is evident in Figure 9A, by the appearance of green cells at 18–30 h post-infection (hpi) in spheroids infected at an MOI of 10 but detected at 12 hpi when infected at an MOI of 50. Thus, the efficiency of infection of 22Rv1 cells in spheroid cultures differs substantially from cell monolayers. Real-time recordings of loss of TRII showed that cell killing was delayed at an MOI of 10 (Figure 9C), consistent with the images showing low infectivity at this MOI. However, loss of TRII was increased by P/V/F infection at a MOI of 50 and did not further increase at MOIs that were above that. In the spheroid cultures infected with the P/V/F mutant (Figure 9), at 60 hpi the P/V/F mutant causes complete spheroid reduction, as evidenced by the lack of detection of red nuclear fluorescence in Figure 9A,B.

Media from spheroids infected at MOIs of 10 and 50 were harvested and virus titers were determined by plaque assay (Supplementary Figure S3A). Virus titers from infected spheroids were similar, at ~10^6^ pfu/mL. Flow cytometry to quantify the percentage of GFP+ cells from spheroids infected at a MOI of 10, showed ~60% GFP+ infected cells (Supplementary Figure S3B). 

Taken together, these data indicate that the P/V/F mutant has the ability to efficiently induce cell killing of 22Rv1 cancer cells in a spheroid tumor model, however, the kinetics of infection and cell killing is slower compared to cell monolayer cultures. 

## 3. Discussion

An important factor in the development of oncolytic viruses for tumor treatment is in uncovering mechanisms and factors that dictate specificity for infection and killing of cancer versus normal cells. Here, we have examined the interactions of three PIV5-based viruses with tumor (22Rv1) and benign, non-tumorigenic (BPH-1) prostate cell lines. The WT PIV5 is not considered a likely oncolytic vector candidate, since it is largely non-cytopathic in most human cell lines. By contrast, the PIV5 Le mutant has been shown to induce apoptosis in the lung cancer cell lines [27] through mechanisms that are activated by viral dsRNA, and thus, this virus has at least some potential properties of oncolytic viruses. Importantly though, our studies shown here reveal that the PIV5 Le mutant is not limited in spread through benign BPH-1 cells. Both WT and Le mutant viruses contain a functional V protein, which limits IFN-I production as well as block IFN-I signaling [9]. Thus, in the case of Le mutant, the presence of a functional V protein in Le mutant-infected cells removes the important selectivity for benign versus cancer cells. In contrast, we show here, that the P/V/F mutant (which is defective in blocking IFN-I induction and signaling) was highly restricted for growth in BPH-1 cells, and this restriction could be relieved by the inhibition of extracellular IFN-I. Taken together, our results show that the status of the P/V gene is an important factor in the selectivity of PIV5 vectors for cancer versus benign cells in vitro, and support further development of the P/V/F mutant as a potential oncolytic virus candidate.

Our data shows that P/V/F mutant growth in benign cells is restricted upon a low MOI infection, while still effectively spreading in tumor cells, which is an ideal scenario for a potential oncolytic viral candidate. The limited spread of the P/V/F mutant through benign BPH-1 cells was not due to an inherent inability to infect and replicate in these cells. To further characterize a fully infected population of both benign and cancer cells, high MOI infections with the P/V/F mutant were performed, which resulted in similar infection and killing of both 22Rv1 and BPH-1 cell lines. Interestingly, caspase-8, -9, and -3/7 levels induced by P/V/F mutant infection were significantly higher in 22Rv1 cells compared to BPH-1 cells at late times post-infection. In particular, the difference in induction of initiator (caspase-8, and -9) caspases was higher than that of effector caspase-3/7, suggesting that 22Rv1 cells may be primed for activation of apoptosis compared to benign cells. One possible approach to further improve selectivity for cancer versus benign cells could include using oncolytic viruses in combination with drugs that target cellular inhibitors of apoptosis (IAPs, e.g., Survivin, XIAP), as shown in our prior work with PIV5 infected respiratory cells [30].

The hyperfusogenic P/V/F mutant was able to induce syncytia formation in 22Rv1 cells, however, this was not observed to any significant extent in infected BPH-1 cells. Prior work has shown that the plasma membrane composition can influence PIV5-induced cell–cell fusion [31,32], suggesting that the composition of plasma membranes in benign vs. tumor cells could be one possibility for differential syncytia formation following P/V/F infection in the two cell lines. Typically, the plasma membrane of non-cancer cells is characterized by lipid asymmetry between the outer and inner membrane leaflets. This includes enrichment of certain lipids, such as phosphatidylserine (PS) and phosphatidylethanolamine (PE) in the inner membrane leaflet, and the presence of lipids, such as phosphatidylcholine (PC) and sphingomyelin (SM) in the outer membrane leaflet [33]. By contrast, in some cancer cells, this asymmetrical distribution is disrupted, resulting in more homogeneity in lipids between bilayers. In addition, some cancer cells have been shown to have upregulated levels of PS on the outer membrane leaflet, while some cancer cells show low levels of SM compared to non-tumor cells [33,34,35]. Prostate cancer progression has been linked to high-level expression of lipid metabolism genes (e.g., OLR1, GLRX and SNAP23) which could contribute to the dysregulation of lipid distribution in the tumor cell membrane [36]. For example, lipidomic profiling of a panel of prostate cancer cells showed that 22Rv1 cells had a high abundance of PC, PE and SM [37], which may account for the differences in syncytia formation of 22Rv1 vs. BPH-1 cells upon infection with the P/V/F mutant. Further investigation is required to determine if differences in plasma membrane composition could act as an additional mechanism of specificity for the P/V/F mutant to target only tumor cells. 

The tumor microenvironments comprise complex interactions between tumor cells, healthy cells and various cytokines, such as IFN-I [10]. To test a working model, co-culture experiments were performed to simulate the interface between a mixed population of solid tumors (22Rv1) and benign cells (BPH-1). Upon a low MOI infection in the co-culture, the restriction of the P/V/F mutant was exhibited within the intact BPH-1 cell population, but not within the intact 22Rv1 cell population. This observation further strengthens the P/V/F mutant as a potential oncolytic viral candidate, as it indicates the mutant has specificity towards cancer cells in a mixed population with benign cells. 

There is a growing interest in three-dimensional versus two-dimensional monolayer cell cultures as a more representative model of in vivo tumor microenvironment, since three-dimensional cultures have the potential to simulate some important factors, such as cell-cell interactions, cell architecture, metabolism and morphology [38,39,40]. Given that the P/V/F mutant induces syncytia in monolayer cultures of 22Rv1 cells, we reasoned that three-dimensional spheroid cultures would be very sensitive to virus-mediated killing due to the ability of the virus to spread from cell to cell and kill by fusion. While the P/V/F mutant was able to infect spheroid cultures of 22Rv1 cells, the efficiency of infection and the kinetics of cell killing were delayed compared to monolayer cultures and required a higher MOI to initiate the infection. Future studies will determine the various factors that could lead to a lower sensitivity of spheroid cultures versus monolayer cultures to P/V/F infection and killing. This could include the possible varying landscape of (non-IFN-I) anti-viral responses within the spheroid, the lipid composition within layers of cells in the spheroids, and restricted access to the virus receptor sialic acid [41]. Further dissecting of the role of syncytia formation or fusion, along with IFN-I competency upon infections in three-dimensional models, will help us better understand the effect of an oncolytic virus in a complex tumor microenvironment. 

We recognize that a limitation of this study involves the comparison of P/V/F mutant properties in a representative benign (BPH-1) and tumor cell line (22Rv1), which are also cell lines of different origins. Our future studies will focus on characterizing the P/V/F mutant in additional benign, non-tumorigenic cell lines (RWPE-1, pRNS-1-1). We are also planning to expand our studies to include patient-derived healthy vs. tumor cells to observe the specificity of the virus within the context of single-source donors. Overall, this study indicated the clinical implications of utilizing the P/V/F mutant for tumor therapy. Our findings indicate that the P/V/F mutant is selective for tumors that have dysfunctional IFN-I machinery. Therefore, it may be possible to screen tumors for IFN-I competence and functionality before administration of the P/V/F mutant, or other IFN-I sensitive viral vectors. In the future, personalized medicine would enable patients to be tested for tumor type and IFN-I status, and the oncolytic virus to be used for treatment will need to be tailored accordingly.

## 4. Materials and Methods

### 4.1. Cells, Viruses and Plaque Assays

Benign prostate cell line (BPH-1) and prostate cancer cell line (22Rv1) were kindly provided by R. Chakrabarti (University of Central Florida, Orlando, FL, USA). A549, Vero, MDBK, and CV-1 cells were procured from ATCC (American Type Culture Collection). BPH-1, A549 alveolar adenocarcinoma cells, Vero, Madin-Darby bovine kidney (MDBK) and African green monkey kidney fibroblasts (CV-1) cells were maintained in Dulbecco’s modified Eagle medium (DMEM) supplemented with 10% heat-inactivated fetal bovine serum (HI-FBS) (Gibco™, Thermo Fisher Scientific, Waltham, MA, USA). HEKn (Normal Human Epidermal Keratinocytes neonatal) cells were procured from Lifeline^®^ Cell Technology (Frederick, MD, USA) and cultured in Lifeline^®^ DermaLife K Medium. The 22Rv1 cells were grown in Roswell Park Memorial Institute-1640 medium (RPMI-1640) supplemented with 10% HI-FBS. HEK-Blue™ IFN-α/β cells (InvivoGen, San Diego, CA, USA) were grown in DMEM supplemented with 10% HI-FBS, 30 µg/mL Blasticidin and 10 µg/mL Zeocin. Nuc Light Red 22Rv1 cells (22Rv1-NLR) were generated by transduction of 22Rv1 cells with lentivirus expressing a red fluorescent protein, purchased from Incucyte (Sartorius, Bohemia, NY, USA). The 22Rv1-NLR cells were cultured in RPMI-1640 supplemented with 10% HI-FBS and 3 µg/mL Puromycin. All cell lines were maintained at 37 °C and under 5% CO_2_ atmospheric conditions. 

WT PIV5 expressing GFP was recovered from a cDNA plasmid, as previously described [29]. The generation of PIV5 hyperfusogenic mutant P/V/F, Le mutant and P/V mutant have been described previously [23,27,28]. Stocks of WT PIV5 virus were grown in MDBK cells while mutants were grown in Vero cells. Infections were carried out by incubating cells with the virus in DMEM supplemented with 10% Bovine Serum Albumin (BSA, Gibco™). After one hour of incubation, virus media was removed, and cells were washed with phosphate-buffered saline (PBS) and replaced with DMEM supplemented with 2% HI-FBS. Viral titers were determined by carrying out plaque assays on CV-1 cells, as previously described [23].

### 4.2. Co-Culture Studies

The 20,000 BPH-1 and 40,000 22Rv1 cells were stained with the CellTrace™ Violet and CellTrace™ Far Red Cell Proliferation Kits (Invitrogen, Thermo Fisher Scientific), as per manufacturer’s instructions, at working concentrations of 5 µM and 2 µM, respectively. Cells were either seeded alone or together in a culture of RPMI-1640, supplemented with 10% HI-FBS in a 24-well plate format. The cells were allowed to settle for 1 day (D) at 37 °C and under 5% CO_2_ atmospheric conditions and subsequently infected at an MOI of 0.01. Flow cytometry was then performed at 3D pi to determine the percentage of stained cells; CellTrace™ Violet stained BPH-1 cells were detected in the PB450 channel (PB450+ cells), while CellTrace™ Far Red stained 22Rv1 cells were detected in the APC channel (APC+ cells). To determine the percentage of infected cells, stained BPH-1 (PB450+GFP+) and 22Rv1 (APC+GFP+) cells were detected in the FITC/GFP channel. 

### 4.3. IFN-I Treatment and IFN-β Antibody Neutralization

Universal IFN-I (PBL Assay Science, Piscataway, NJ, USA) is a hybrid of human IFN-I proteins (Human Interferon Alpha A/D [BglII]), used to signal through the IFN-I receptor as a hybrid of IFN-α and IFN-β. BPH-1 and 22Rv1 cells were treated with universal IFN-I at the indicated concentrations (0, 20, 200, and 2000 units/mL) in DMEM supplemented with 2% HI-FBS. In the IFN-β neutralization experiment performed, a neutralizing antibody (Ab) against IFN-β (MilliporeSigma, Burlington, MA, USA) was used at a dilution of 1:20 while TNF-α Ab (Biosource, Thermo Fisher Scientific) was used at a final concentration of 10 µg/mL. Neutralizing Abs were added at the indicated concentrations directly into DMEM supplemented with 2% HI-FBS, after performing the virus infection.

### 4.4. Flow Cytometric Analysis

Mock or virus-infected 22Rv1 cells were trypsinized, while BPH-1 cells were detached using Accutase™ (MilliporeSigma). Due to longer detachment times for BPH-1 cells, Accutase™ was utilized, to avoid the potentially harsh effect of trypsin. Cells were then quenched in DMEM supplemented with HI-FBS and washed with PBS. Flow cytometry was performed using the FACS Canto (BD Biosciences, San Jose, CA, USA) or the CytoFLEX (Beckman Coulter, Brea, CA, USA), and 10,000 independent events were recorded. Results were analyzed using the Flowlogic software (for FACS Canto) or CytExpert (for CytoFLEX).

### 4.5. Cell Viability, Death and Caspase Assays

Propidium Iodide (PI) (BD Pharmingen™, BD Biosciences) was used to quantify non-viable cells. An amount of 1 µL of PI dye was added to the cell suspension prior to performing flow cytometry. For caspase assays, 5000 22Rv1 cells or 2000 BPH-1 cells were seeded in white 96-well plates (Corning, NY, USA). Caspase-Glo^®^ 8, Caspase-Glo^®^ 9, and Caspase-Glo^®^ 3/7 Assay systems (Promega, Madison, WI, USA) were carried out as per the manufacturer’s instructions. Luminescence readings were taken 90 min (min) post-addition of the reagent. Readings were normalized to that of mock-infected cells and data are expressed as fold of virus-infected cells over mock-infected cells.

### 4.6. Spheroid Generation, Infection and Analysis

Spheroids were generated by plating 22Rv1-NLR cells at 1000 cells/well in a 96-well ultra low attachment (ULA) plate (Costar, Corning), centrifuged for 20 min at 130× *g,* and cultured for 4 days at 37 °C and under 5% CO_2_ atmospheric conditions. Spheroids were infected by adding an appropriate volume of the virus to each well, in replicates of 6. Plates were then incubated in the Incucyte SX5 Live-Cell Analysis system (Sartorius) at 37 °C and under 5% CO_2_ atmospheric conditions for 4 days. Images were captured by the Incucyte every 6 h under 10× magnification in phase, red and green fluorescent channels. The Incucyte measures red calibrated units (RCU) in the field of view (a spheroid) and then calculates the total red integrated intensity (TRII) by measuring the total RCU of the spheroid per image by the formula (RCU × μm^2^/Image) [38]. TRII was monitored over the indicated time course and each data point was expressed as a percentage of the initial value at time 0 (TRII/TRII^t0^).

To determine viral titers, spheroids were infected by adding an appropriate volume of virus to each well and incubating overnight at 37 °C and under 5% CO_2_ atmospheric conditions. Infected spheroids were washed with PBS and replaced with DMEM supplemented with 2% HI-FBS. The supernatant was collected at 2D post-replacement to quantify infectious units by plaque assays. Additionally, infected spheroids were then processed for flow cytometry to determine the percentage of GFP+ infected cells. 

### 4.7. IFN-I Detection Assay

To inactivate the virus, supernatant from infected cells was treated with 1N HCl for 30 min at room temperature and subsequently titrated with 1N NaOH to achieve neutralization of acid. The resulting acid-neutralized media was used to treat HEK-Blue™ IFN-α/β cells overnight, as per the manufacturer’s instructions. A standard curve with known concentrations of universal IFN-I was also generated. The secreted alkaline phosphatase (SEAP) produced in response to either the stimulation with acid-neutralized media or standard IFN-I treatment was measured by the addition of QUANTI-Blue™ (Invivogen). Absorbance was read at 650 nm at 15 min intervals for 1 hr. Amounts of IFN-I in the collected media were determined using the standard curve and expressed as units/mL.

### 4.8. Reverse Transcription and Real-Time PCR

Total cellular RNA from 10^5^ mock or virus-infected cells was isolated using TRIzol (Invitrogen, Thermo Fisher Scientific). An amount of 1µg of total RNA was used to obtain cDNA by utilizing the TaqMan^®^ Reverse Transcription reagents (Applied Biosystems, Thermo Fisher Scientific), as per the manufacturer’s instructions. Quantitative real-time PCR was performed using Bio-Rad CFX Connect Real-Time (CFX96™) and Fast SYBR^®^ FAST Green Master Mix (Applied Biosystems, Thermo Fisher Scientific). Primers used include β-actin forward 5′-GATCATTGCTCCTCCTGAGC-3′, and β-actin reverse 5′-ACTCCTGCTTGCTGATCCAC-3′. OAS2 forward: 5′-AGAAGCTGGGTTGGTTTATC-3′, and OAS2 reverse 5′- GACGTCACAGATGGTGTTC-3′. IFIT1 forward and reverse primers were obtained from a previous study [42], as were the TLR3 forward and reverse primers [43]. Primers were used at a final concentration of 2 µM, with the following amplification conditions for qRT-PCR: 95 °C for 5 min, 95 °C for 10 s followed by 30 s at 61.6 °C for 34 cycles, 95 °C for 15 s. Melt curve analysis was performed as per CFX96™ protocol from 65.0–95 °C with 0.5 increments. The threshold cycle (Ct) for the gene of interest was normalized to the housekeeping gene β-actin and was set to the threshold Ct value of 38. Relative gene expression was determined by comparing 2^(-Avg.(Delta(Ct)) values generated by the RT^2^ Profiler PCR array analysis software, a web-based service for assaying multiple genes (SABiosciences, Qiagen, Maryland, USA) [44]. This software also provided statistical analyses, by generating *p*-values using a Student’s *t*-test. 

### 4.9. Fluorescence Microscopy

Before imaging, at indicated times post-infection, infection media was replaced with PBS. Fluorescence microscopy was then performed using a Zeiss Axiovert fluorescence microscope under a 10× or 20× lens (Carl Zeiss Microscopy, LLC, White Plains, NY, USA). Exposure time for the bright field (BF) was 46ms and was adjusted for fluorescence (FL).

### 4.10. Figures and Statistics

Averages and standard deviations were generated using GraphPad^®^ (GP) Prism software. The *p*-values were generated using two-way ANOVA or GP’s Student’s *t*-test, with α = 0.05. In all figures, *, **, ***, and **** indicating *p*-values < 0.033, <0.002, <0.0002 and <0.0001, respectively (*p*-value style was set to GP). Graphs were generated using GP Prism software.

## Data Availability

Data availability is at request.

## Supplementary Materials

The following supporting information can be downloaded at: https://www.mdpi.com/article/10.3390/pathogens11050493/s1, Figure S1: Cytopathic effect following low MOI infection with P/V/F mutant in 22Rv1 cells; Figure S2: Differential infection outcomes in Human Epidermal Keratinocytes (HEKn) with control WT virus and P/V/F mutant; Figure S3: Growth of P/V/F mutant in 3-dimensional spheroid tumor model.

## References

[1] American Cancer Society Cancer Statistics Center. https://www.cancer.org/cancer/prostate-cancer/about/key-statistics.html.

[2] Andtbacka R.H., Kaufman H.L., Collichio F., Amatruda T., Senzer N., Chesney J., Delman K.A., Spitler L.E., Puzanov I., Agarwala S.S. (2015). Talimogene Laherparepvec Improves Durable Response Rate in Patients with Advanced Melanoma. J. Clin. Oncol..

[3] Chiocca E.A. (2002). Oncolytic viruses. Nat. Rev. Cancer.

[4] Bell J.C., Lichty B., Stojdl D. (2003). Getting oncolytic virus therapies off the ground. Cancer Cell.

[5] Chiocca E.A., Rabkin S.D. (2014). Oncolytic viruses and their application to cancer immunotherapy. Cancer Immunol. Res..

[6] Bateman A.R., Harrington K.J., Kottke T., Ahmed A., Melcher A.A., Gough M.J., Linardakis E., Riddle D., Dietz A., Lohse C.M. (2002). Viral fusogenic membrane glycoproteins kill solid tumor cells by nonapoptotic mechanisms that promote cross presentation of tumor antigens by dendritic cells. Cancer Res..

[7] Melcher A., Gough M., Todryk S., Vile R. (1999). Apoptosis or necrosis for tumor immunotherapy: What’s in a name?. J. Mol. Med..

[8] Errington F., Jones J., Merrick A., Bateman A., Harrington K., Gough M., O’Donnell D., Selby P., Vile R., Melcher A. (2006). Fusogenic membrane glycoprotein-mediated tumour cell fusion activates human dendritic cells for enhanced IL-12 production and T-cell priming. Gene Ther..

[9] Randall R.E., Goodbourn S. (2008). Interferons and viruses: An interplay between induction, signalling, antiviral responses and virus countermeasures. J. Gen. Virol..

[10] Aricò E., Castiello L., Capone I., Gabriele L., Belardelli F. (2019). Type I Interferons and Cancer: An Evolving Story Demanding Novel Clinical Applications. Cancers.

[11] Matveeva O.V., Chumakov P.M. (2018). Defects in interferon pathways as potential biomarkers of sensitivity to oncolytic viruses. Rev. Med. Virol..

[12] Asada T. (1974). Treatment of human cancer with mumps virus. Cancer.

[13] Fiola C., Peeters B., Fournier P., Arnold A., Bucur M., Schirrmacher V. (2006). Tumor selective replication of Newcastle disease virus: Association with defects of tumor cells in antiviral defence. Int. J. Cancer.

[14] Pecora A.L., Rizvi N., Cohen G.I., Meropol N.J., Sterman D., Marshall J.L., Goldberg S., Gross P., O’Neil J.D., Groene W.S. (2002). Phase I trial of intravenous administration of PV701, an oncolytic virus, in patients with advanced solid cancers. J. Clin. Oncol..

[15] Myers R., Greiner S., Harvey M., Soeffker D., Frenzke M., Abraham K., Shaw A., Rozenblatt S., Federspiel M.J., Russell S.J. (2005). Oncolytic activities of approved mumps and measles vaccines for therapy of ovarian cancer. Cancer Gene Ther..

[16] Peng K.W., TenEyck C.J., Galanis E., Kalli K.R., Hartmann L.C., Russell S.J. (2002). Intraperitoneal therapy of ovarian cancer using an engineered measles virus. Cancer Res..

[17] Galanis E., Hartmann L.C., Cliby W.A., Long H.J., Peethambaram P.P., Barrette B.A., Kaur J.S., Haluska P.J., Aderca I., Zollman P.J. (2010). Phase I trial of intraperitoneal administration of an oncolytic measles virus strain engineered to express carcinoembryonic antigen for recurrent ovarian cancer. Cancer Res..

[18] Didcock L., Young D.F., Goodbourn S., Randall R.E. (1999). The V protein of simian virus 5 inhibits interferon signalling by targeting STAT1 for proteasome-mediated degradation. J. Virol..

[19] Sun M., Rothermel T.A., Shuman L., Aligo J.A., Xu S., Lin Y., Lamb R.A., He B. (2004). Conserved cysteine-rich domain of paramyxovirus simian virus 5 V protein plays an important role in blocking apoptosis. J. Virol..

[20] Lamb R.A., Parks G.D., Knipe D.M., Howley P.M., Griffin D.E., Lamb R.A., Martin M.A., Roizman B., Straus S.E. (2007). Paramyxoviridae: The viruses and their replication. Fields Virology.

[21] Horvath C.M. (2004). Weapons of STAT destruction. Interferon evasion by paramyxovirus V protein. Eur. J. Biochem..

[22] Dillon P.J., Wansley E.K., Young V.A., Alexander-Miller M.A., Parks G.D. (2006). Exchange of P/V genes between two non-cytopathic simian virus 5 variants results in a recombinant virus that kills cells through death pathways that are sensitive to caspase inhibitors. J. Gen. Virol..

[23] Wansley E.K., Parks G.D. (2002). Naturally occurring substitutions in the P/V gene convert the noncytopathic paramyxovirus simian virus 5 into a virus that induces alpha/beta interferon synthesis and cell death. J. Virol..

[24] Wansley E.K., Dillon P.J., Gainey M.D., Tam J., Cramer S.D., Parks G.D. (2005). Growth sensitivity of a recombinant simian virus 5 P/V mutant to type I interferon differs between tumor cell lines and normal primary cells. Virology.

[25] Young V.A., Dillon P.J., Parks G.D. (2006). Variants of the paramyxovirus Simian virus 5 with accelerated or delayed viral gene expression activate proinflammatory cytokine synthesis. Virology.

[26] Gainey M.D., Dillon P.J., Clark K.M., Manuse M.J., Parks G.D. (2008). Paramyxovirus—Induced shutoff of host and viral protein synthesis: Role of the P and V proteins in limiting PKR activation. J. Virol..

[27] Manuse M.J., Parks G.D. (2009). Role for the paramyxovirus genomic promoter in limiting host cell antiviral responses and cell killing. J. Virol..

[28] Gainey M.D., Manuse M.J., Parks G.D. (2008). A hyperfusogenic F protein enhances the oncolytic potency of a paramyxovirus simian virus 5 P/V mutant without compromising sensitivity to type I interferon. J. Virol..

[29] He B., Paterson R.G., Ward C.D., Lamb R.A. (1997). Recovery of infectious SV5 from cloned DNA and expression of a foreign gene. Virology.

[30] Fox C.R., Parks G.D. (2018). Parainfluenza Virus Infection Sensitizes Cancer Cells to DNA-Damaging Agents: Implications for Oncolytic Virus Therapy. J. Virol..

[31] Roos D.S., Duchala C.S., Stephensen C.B., Holmes K.V., Choppin P.W. (1990). Control of virus-induced cell fusion by host cell lipid composition. Virology.

[32] Yao Q., Hu X., Compans R.W. (1997). Association of the parainfluenza virus fusion and hemagglutinin-neuraminidase glycoproteins on cell surfaces. J. Virol..

[33] Lladó V., López D.J., Ibarguren M., Alonso M., Soriano J.B., Escribá P.V., Busquets X. (2014). Regulation of the cancer cell membrane lipid composition by NaCHOleate: Effects on cell signaling and therapeutical relevance in glioma. Biochim. Biophys. Acta.

[34] Tan L.T., Chan K.G., Pusparajah P., Lee W.L., Chuah L.H., Khan T.M., Lee L.H., Goh B.H. (2017). Targeting Membrane Lipid a Potential Cancer Cure?. Front. Pharm..

[35] Zalba S., Ten Hagen T.L. (2017). Cell membrane modulation as adjuvant in cancer therapy. Cancer Treat. Rev..

[36] Hirsch H.A., Iliopoulos D., Joshi A., Zhang Y., Jaeger S.A., Bulyk M., Tsichlis P.N., Shirley Liu X., Struhl K. (2010). A transcriptional signature and common gene networks link cancer with lipid metabolism and diverse human diseases. Cancer Cell.

[37] Sorvina A., Bader C.A., Caporale C., Carter E.A., Johnson I.R.D., Parkinson-Lawrence E.J., Simpson P.V., Wright P.J., Stagni S., Lay P.A. (2018). Lipid profiles of prostate cancer cells. Oncotarget.

[38] Varudkar N., Oyer J.L., Copik A., Parks G.D. (2021). Oncolytic parainfluenza virus combines with NK cells to mediate killing of infected and non-infected lung cancer cells within 3D spheroids: Role of type I and type III interferon signaling. J. Immunother. Cancer.

[39] Jensen C., Teng Y. (2020). Is It Time to Start Transitioning from 2D to 3D Cell Culture?. Front. Mol. Biosci..

[40] Berg D.R., Offord C.P., Kemler I., Ennis M.K., Chang L., Paulik G., Bajzer Z., Neuhauser C., Dingli D. (2019). In vitro and in silico multidimensional modeling of oncolytic tumor virotherapy dynamics. PLoS Comput. Biol..

[41] Tong J.G., Valdes Y.R., Barrett J.W., Bell J.C., Stojdl D., McFadden G., McCart J.A., DiMattia G.E., Shepherd T.G. (2015). Evidence for differential viral oncolytic efficacy in an in vitro model of epithelial ovarian cancer metastasis. Mol. Ther. Oncolytics.

[42] Madigan A.A., Sobek K.M., Cummings J.L., Green W.R., Bacich D.J., O’Keefe D.S. (2012). Activation of innate anti-viral immune response genes in symptomatic benign prostatic hyperplasia. Genes Immun..

[43] González-Reyes S., Marín L., González L., González L.O., del Casar J.M., Lamelas M.L., González-Quintana J.M., Vizoso F.J. (2010). Study of TLR3, TLR4 and TLR9 in breast carcinomas and their association with metastasis. BMC Cancer.

[44] Li Y., Kakinami C., Li Q., Yang B., Li H. (2013). Human apolipoprotein A-I is associated with dengue virus and enhances virus infection through SR-BI. PLoS ONE.

