# Supplementary material for: Differential In Vitro Growth and Cell Killing of Cancer versus Benign Prostate Cells by Oncolytic Parainfluenza Virus
Source: Pathogens. 2022 Apr 21;11(5):493. doi: 10.3390/pathogens11050493 (PMC9147676; doi:10.3390/pathogens11050493)
Supplement: Supplementary file 1 [file pathogens-11-00493-s001.zip › pathogens-1616449-supplementary.pdf]

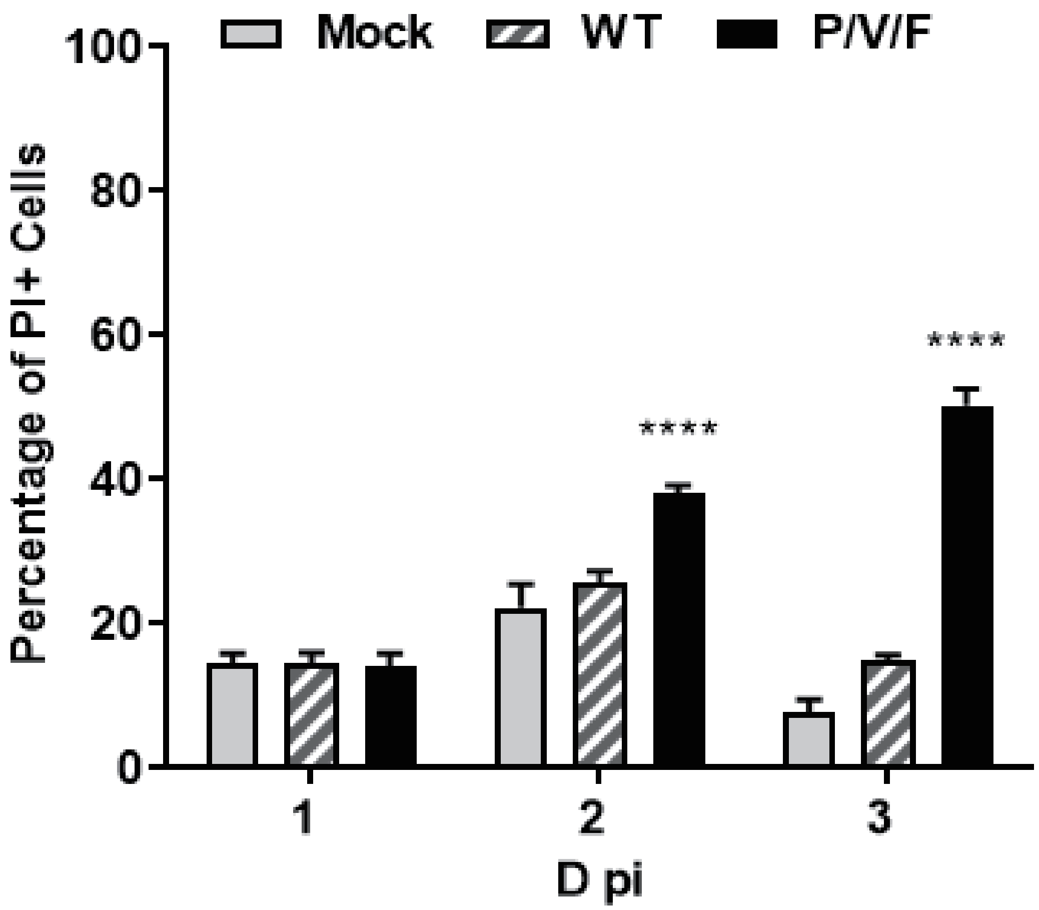

Supplemental Figure S1. Cytopathic effect following low MOI infection with P/V/F mutant in 22Rv1 cells. 22Rv1 cells were infected at a MOI of 0.05 with PIV5 WT and P/V/F mutant and flow cytometry was performed at indicated time points to determine percentage of PI+ cells. Values are the mean of three samples, with error bars representing standard deviation. **** indicating p-value <0.0001.

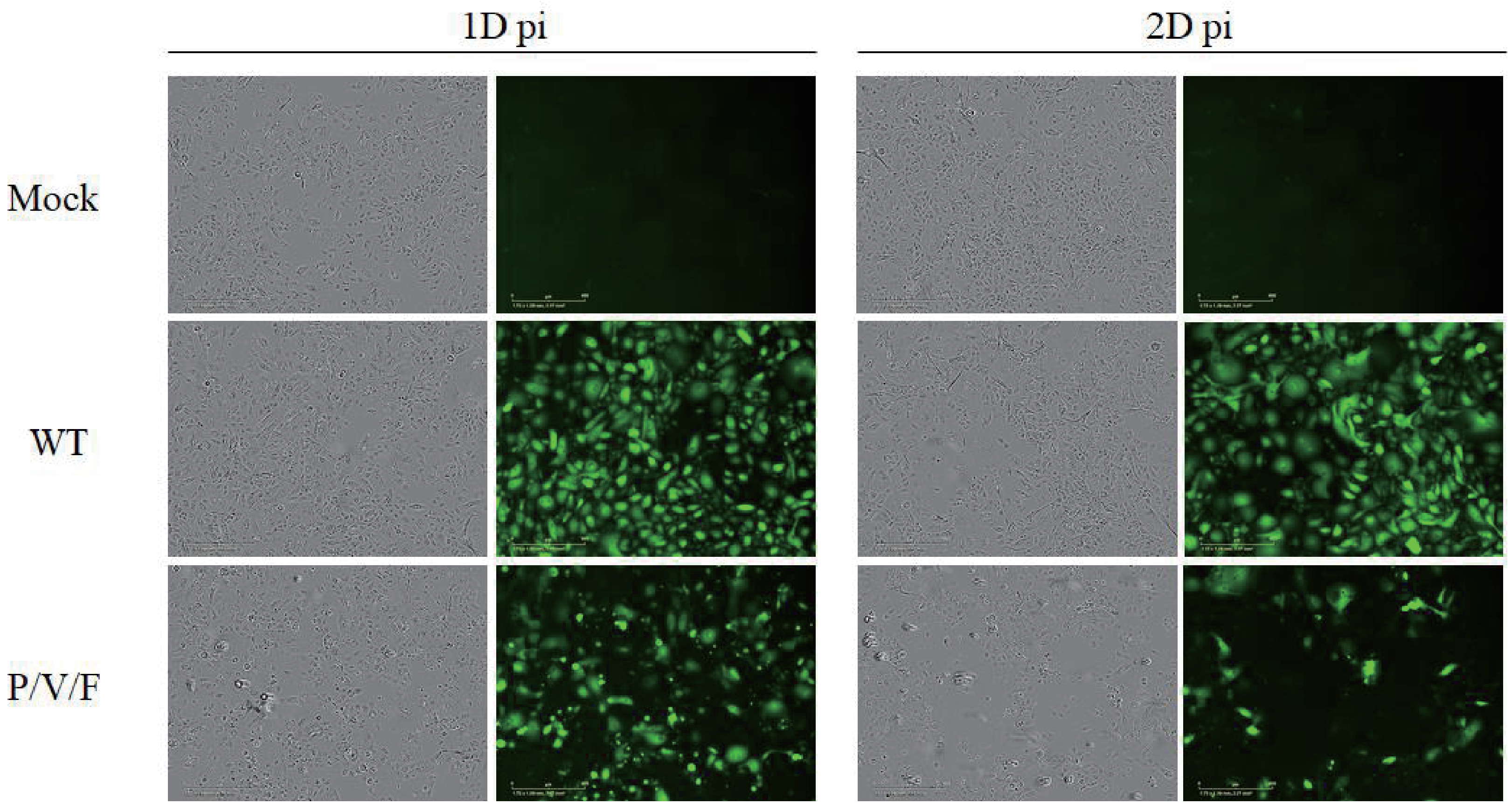

Supplemental Figure S2. Differential infection outcomes in Human Epidermal Keratinocytes (HEKn) with control WT virus and P/V/F mutant. HEKn cells were either mock infected, infected with WT control virus, or P/V/F mutant at a MOI of 10. At the indicated time points, Brightfield (BF) and Fluorescence (FL) images were taken under 10x magnification.

A.

B.

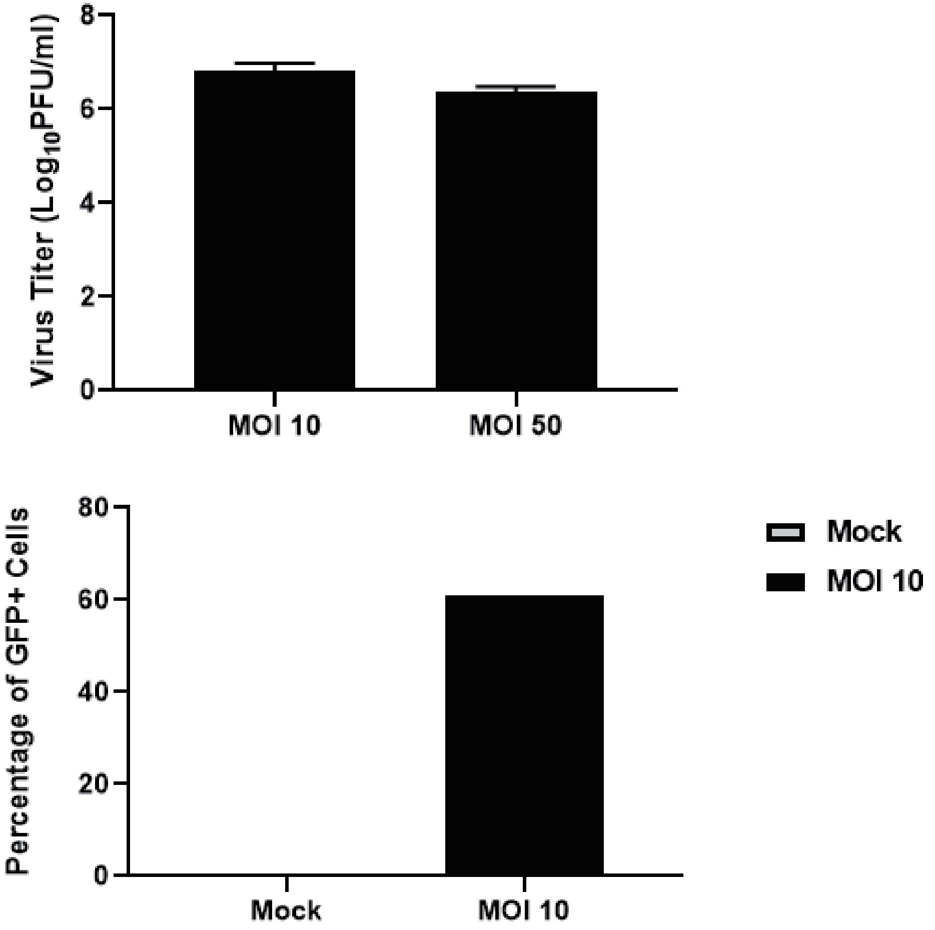

Supplemental Figure S3. Growth of P/V/F mutant in 3-dimensional spheroid tumor model. Spheroid cultures of 22Rv1-NLR cells were infected overnight with P/V/F mutant at MOIs of 10 and 50, washed and replaced with fresh culture media. At 2D post replacement (A) supernatant from infected spheroids was harvested and titers of infectious virus units were determined by plaque assays. Additionally, (B) flow cytometry was performed to determine percentage of GFP+ cells from spheroids infected at MOI of 10.
